# Effects of temperature and host stage on the parasitization rate and offspring sex ratio of *Aenasius bambawalei* Hayat in *Phenacoccus solenopsis* Tinsley

**DOI:** 10.7717/peerj.1586

**Published:** 2016-01-14

**Authors:** Juan Zhang, Jun Huang, Yaobin Lu, Tianfeng Xia

**Affiliations:** 1 Flower Research and Development Centre, Zhejiang Academy of Agricultural Sciences, Hangzhou, Zhejiang Province, China; 2 State Key Laboratory Breeding Base for Zhejiang Sustainable Pest and Disease Control, Institute of Plant Protection and Microbiology, Zhejiang Academy of Agricultural Sciences, Hangzhou, Zhejiang Province, China

**Keywords:** *Phenacoccus solenopsis*, **Aenasius bambawalei**, Heat stress, Host stage, Parasitization rate, Offspring sex ratio

## Abstract

Temperature and host stage are important factors that determine the successful development of parasitoids. *Aenasius bambawalei* Hayat (Hymenoptera: Encyrtidae) is a primary parasitoid of the newly invasive mealybug, *Phenacoccus solenopsis* Tinsley (Hemiptera: Pseudococcidae). The effects of temperature on the parasitic characteristics of *A. bambawalei* have seldom been investigated. In the study, we explored the effects of temperature, exposure time, and host stage on the parasitization rate and offspring sex ratio (female to male) of *A. bambawalei* under laboratory conditions. The laboratory results showed that the successful parasitization rate of *A. bambawalei* increased with higher temperatures and older host stages. When the parasitoids were exposed to 36 °C for 24 h, the parasitization rate of female adults (52%) was nearly two times that of 3rd instar nymphs. Additionally, heat stress duration and host stage resulted in an increase in the offspring sex ratio of *A. bambawalei*. When *A. bambawalei* was exposed to 36 °C for 24 h, the offspring sex ratio increased dramatically to 81.78% compared with those exposed for 12 h, and it increased to 45.34% compared with those exposed for 16 h. The offspring sex ratio was clearly higher when the host stage was an adult female mealybug Our findings provide important guidance for the mass rearing and field releases of *A. bambawalei* for the management of *P. solenopsis* in the future.

## Introduction

In recent years, the dispersal rate of invasive Hemipteran insect pests into agricultural systems has been doubled, and these pests have become one of the leading causes for the lower production of economic crops worldwide (Ahmed et al., 2011). Therefore, it is necessary to control the dispersal of invasive Hemipteran in the fields. Fortunately, such invasions can be controlled by natural enemies (Gautam et al., 2009). However, the environmental effects on those natural enemies when exposed to new conditions are seldom studied.

The cotton mealybug, *Phenacoccus solenopsis* Tinsley (Hemiptera: Pseudococcidae), has been recently recognized as an aggressive invasive insect pest in Asian countries (Wang, Watson & Zhang, 2010). In these countries, this mealybug caused severe economic damage to cotton and to a wide range of vegetables, horticultural plants and other field crops (Nagrare et al., 2011). **Aenasius bambawalei** Hayat (Hymenoptera: Encyrtidae) plays a significant role in controlling the mealybug population (Hayat, 2009; Ashfaq et al., 2010; Fand, Gautam & Suroshe, 2011). Field investigation showed that the parasitization rate of *A. bambawalei* ranged from 50% to 62% (Dhawan et al., 2007; Prasad et al., 2011; Rishi et al., 2009; Tanwar et al., 2011). *A. bambawalei* is adaptable to strict environmental conditions and survives in a temperature range from 2 °C to 45 °C (Nagrare et al., 2011). Indeed, it is a natural enemy from tropical areas, such as India and Pakistan (Fand & Suroshe, 2015) and China. However, temperature changes may affect its parasitic fitness.

Temperature significantly affects the parasitic characteristics of certain parasitoids (Miller, 1996; Bell et al., 2003). For *Fopius arisanus*, the parasitization rate significantly varied with the tested temperatures: it was the highest at 25 °C, and the lowest at 15 °C. The parasitoid progeny sex ratio was female-biased under all the tested temperatures except with 20 °C (Appiah et al., 2013). The intended sex ratio of female *Trichogramma euproctidis* at a low temperature (14 °C) was similar to that at an intermediate temperature (24 °C). However, physiological constraints prevented the egg fertilization during oviposition, which resulted in more males emerging from eggs than what would be expected (Moiroux, Brodeur & Boivin, 2014). Typically, more males were produced when parents and/or eggs were exposed to low or high temperatures (King, 1987; Colinet & Boivin, 2011). The parasitization rate of *A. bambawalei* depends on the season (Kedar, Saini & Pala, 2012; Nagrare et al., 2011). Parasitic fitness is also, to some extent, related with the host stage. Tahriri et al. (2007) noted that *Aphidius matricariae* showed significant differences in the parasitization rate among *Aphis fabae* of different stages. Parasitoids that parasitize at third-instars or pre-reproductive adult females were characterized by the lowest proportion of males (Chong & Oetting, 2006). Under stationary laboratory conditions, the parasitization rate of *A. bambawalei* in third instar nymphs of *P. solenopsis* was higher than that in other stages (Fand, Gautam & Suroshe, 2011), and the offspring sex ratio (female to male) developed in third instar nymphs was higher than that developed in adult hosts (Abdin et al., 2013). In a pilot study, we found that temperature and host stage could influence the parasitization behaviors of *A. bambawalei*.

To elucidate the effect of temperature and host stage on *Aenasius*, we investigated the effects of temperature, exposure time, and host stages on the parasitization rate and offspring sex ratio. The results will expand our knowledge of this parasitoid with respect to its potential as a biological control agent and as a component of integrated pest management (IPM) programs.

## Materials and Methods

### Insects

No specific permission was required for the study and no endangered or protected species were involved. The initial colony of *P. solenopsis* was isolated from infested *Torenia fournieri* plants (Scrophulariales: Scrophulariaceae) in the suburbs of Hangzhou, Zhejiang Province, China. Then, they were reared on *Gossypium hirsutum* (Malvales: Malvaceae) in a climate chamber (27 ± 1 °C with a photoperiod of 14 L: 10 D, and 70 ± 5% relative humidity -RH) at the Zhejiang Academy of Agricultural Sciences, China. The developmental period of *P. solenopsis* has 4 life stages: first-, second-, and third-instar nymphs and adults (Huang et al., 2012b). Based on previous studies (Fand, Gautam & Suroshe, 2011; Abdin et al., 2012b; Abdin et al., 2013) and on our own pilot study, the optimal host stages (female adults and the third instar nymphs) of *P. solenopsis* were used in the experiments.

*A. bambawalei* originated from a colony of *P. solenops*is that was growing on *Hibiscus rosa-sinensis* plants in the Guangdong Province, China. A laboratory colony was established with *P. solenops*is as hosts in May 2012 and then maintained in a growth chamber at 27 ± 1 °C (which was the most suitable temperature for the mealybug in our pilot study), with a photoperiod of 14 L: 10 D and a RH of 75 ± 5%.

### Rearing of insect colonies

The rearing system was set up as described by Huang et al. (2012a). Briefly, a leaf contained 30 female adults of *P. Solenopsis*in the system. Then, six pairs of newly emerged (1-2 d), and mated adults of *A. bambawalei* were introduced into the rearing system. Finally, the rearing system was reversed on a cup of water to prevent leaves from desiccation. The parasitization rate could be observed when the parasitized mealybugs shed their wax, swelled and hardened into a leathery, brown colored structure called a “mummy” (Abdin et al., 2012a). Adult parasitoids emerged 7 days after the formation of the mummies.

### Determination and calculation of the parasitization rate

To investigate the effects of temperature and host stage on the parasitization rate of *A. bambawalei*, a total of 10 adult females or 15 third instar nymphs of *P. Solenopsis* (the best density of adult female and 3rd instar nymphs to obtain the highest parasitization rate, Huang et al., 2012a) were introduced into the rearing system under no-choice conditions. Because the parasitoid age is important in biological control programs, 1-day-old wasps were used in this study (Kant et al., 2013). Two pairs of mated *A. bambawalei* were introduced into the rearing system with a simple suction device. The pilot experiments performed before this study indicated that the parasitization rate was a linear function of temperature across the exposure and reached the peak value at 36 °C. Therefore, the parasitization rate of the 3rd instar nymph mealybugs was determined in the laboratory at different temperatures (21 °C, 24 °C, 27 °C, 30 °C, 33 °C, 36 °C, and 39 °C) for 1 h, 2 h, 4 h, 8 h, 12 h, 16 h, and 24 h (Fand, Gautam & Suroshe, 2011) to simulate the real temperature conditions present from May to September in Hangzhou, China. Meanwhile, the parasitization rate of the adult female mealybugs was determined with the same range of temperatures as those used for 3rd instar nymphs. At the end of the exposure, the environment was cooled down to 27 °C (the optimal temperature for *P. solenopsis* in our pilot study), and the wasps were removed from the system. Afterwards, *P. solenopsis* was observed every day, and its mummies were recorded during the next 20 days. The encapsulation is an immune response of the host against the intrusion of an external element (Ratcliffe, 1993; Jervis & Copland, 1996). For encyrtid insects that parasitize mealybugs, the host immune responses are poorly documented (Sagarra et al., 2000), especially in the egg and juvenile stages of the mealybug (Blumberg, Klein & Mendel, 1995). In our pilot study, we found the encapsulation of nymphs and adult females as described by Sagarra et al. (2000). Thus, in this study, the ratio of mummies to the total number of *P. solenopsis* adults or nymphs represents the successful parasitization rate. Each treatment included 5 replicates. The stage of *P. solenopsis* parasitized by *A. bambawalei* under a growth system with a photoperiod of L 14: D 10 and with 75 ± 5% RH were used as the untreated control.

### Determination and calculation of offspring sex ratio

*P. solenopsis* parasitized by *A. bambawalei* (i.e., mummies) were obtained from the parasitization experiment and then maintained at 27 °C separately to reveal the effects of temperature and host stage on the offspring sex ratio. The number of female and male adults that emerged was examined once every 12 h. The offspring sex ratio was indicated by the numbers of female to male adults. Each treatment included 5 replicates. Parasitized mummies maintained at the photoperiod of L 14: D 10 and with 75 ± 5% RH were used as the untreated control.

### Data analysis

The effects of temperature and host stage on the successful parasitization rate and offspring sex ratio were analyzed using repeated analysis of variance (ANOVA) measures and illustrated with Sigmaplot version 12.0. A two-way ANOVA was used to test the importance of temperature and host stage on the overall parasitization rate and offspring sex ratio. To determine whether there was an interaction between temperature and host stage, a two-way ANOVA was used to test the difference between the two host stages and the seven different temperatures. Next, multiple comparisons were performed with the Tukey’s test. Then, a paired *t*-test was performed to compare the parasitization rate and the offspring sex ratio of *A. bambawalei* between female adults and 3rd instar nymphs of *P. Solenopsis* as host exposed for 24 h. All the statistical analyses were conducted in SPSS version 14.0 (SPSS Inc., Chicago, IL, USA). The data were expressed as the means ± standard error.

**Figure 1:**
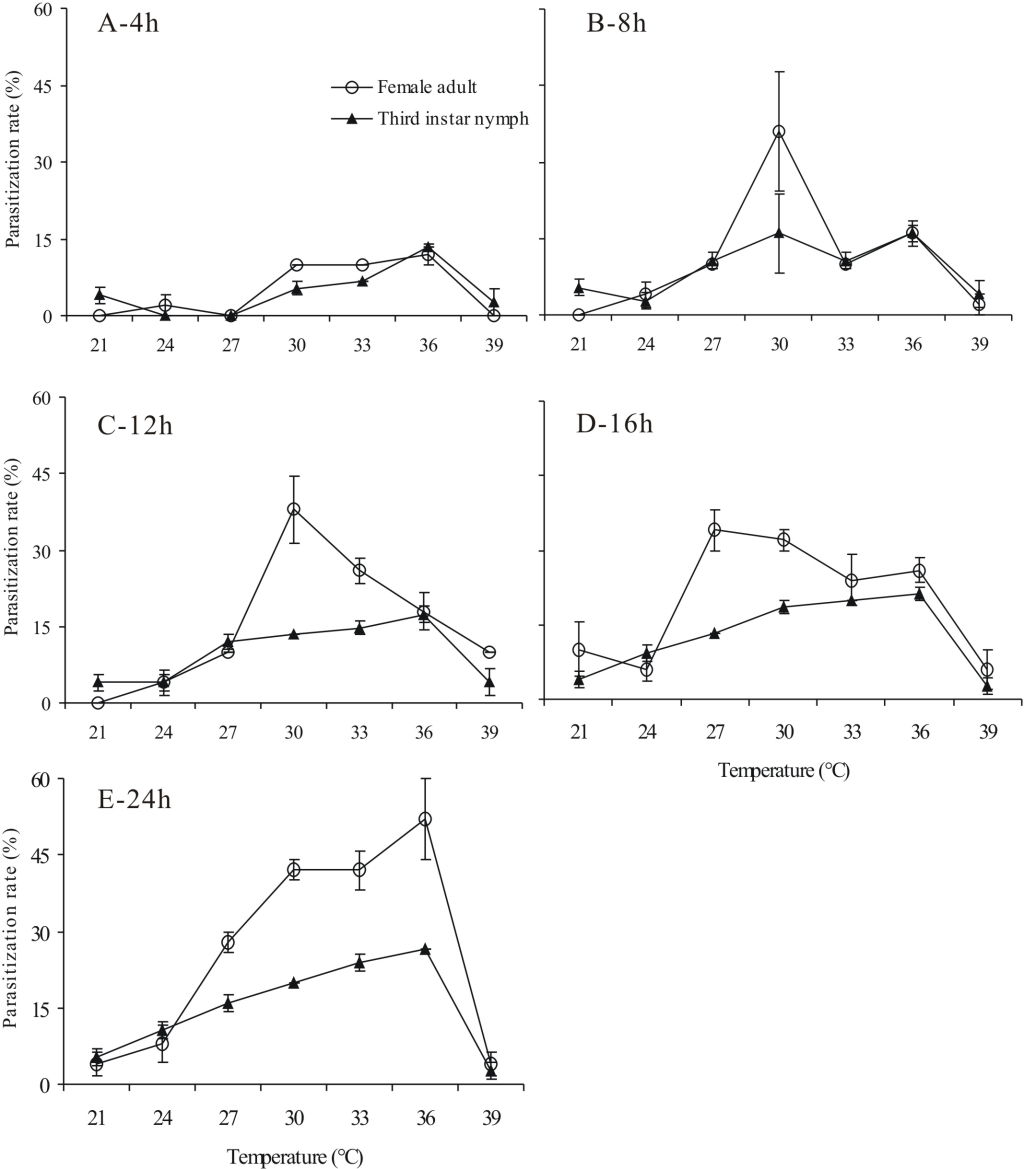
Successful parasitization rate (%) of *Aenasius bambawalei* exposed to temperatures of 21–39 °C for 4 h (A), 8 h (B), 12h (C), 16h (D), and 24 h (E).

**Table 1:** Two-way analysis of variance testing the effect of temperature and host stage on the successful parasitization rate of *Aenasius bambawalei*.

Duration	Source	d*f* (numerator, denominator)	Mean squares	*F*	*P*
4 h	T	6, 69	0.0233	33.375	<0.01
	H	1, 69	0.0001	0.204	0.6535
	T × H	6, 69	0.0025	3.649	<0.01
8 h	T	6, 69	0.0732	8.831	<0.01
	H	1, 69	0.0057	0.691	1.4093
	T × H	6, 69	0.0172	2.073	0.071
12 h	T	6, 69	0.0794	25.068	<0.01
	H	1, 69	0.048	15.167	<0.01
	T × H	6, 69	0.0215	7.913	<0.01
16 h	T	6, 69	0.0878	21.272	<0.01
	H	1, 69	0.0846	20.795	<0.01
	T × H	6, 69	0.0151	3.661	<0.01
24 h	T	6, 69	0.2156	48.503	<0.01
	H	1, 69	0.1991	44.79	<0.01
	T × H	6, 69	0.0337	7.574	<0.01

**Notes.**

T Temperature H Host stage T × H Temperature × Host stage

## Results

### Parasitization rate

Previous results indicated that temperature significantly affect the parasitization rate of the parasitic wasp (Tahriri et al., 2007; Appiah et al., 2013). In our study, the successful parasitization rates increased with higher temperature and with more mature host stages (Fig. 1 and Table 1). A subsequent parasitization rate analysis revealed a more beneficial effect following the treatments of 30, 33, and 36 °C (Fig. 1). Likewise, a significant difference was observed between different host stages, and the parasitization rate depending partly on the time of exposure (Fig. 1). The parasitization rate of adult females exposed for 12 h, 16 h, and 24 h were significantly higher than that of 3rd instar nymphs. In contrast, no difference was detected when adult females and 3rd instar nymphs exposed for 4 h and 8 h. When exposed to 36 °C for 24 h, the parasitization rate of female adults (52%) was nearly two times that of 3rd instar nymphs (26.67%; *t* = 3.166, *P* = 0.034). This result is consistent with the result obtained by Abdin et al. (2012b), but it contradicts that obtained by Fand, Gautam & Suroshe (2011) at 27 °C.

Moreover, we found a significant interaction (*P* < 0.05) between temperature and host stage (Table 1). The highest parasitization rate was detected when adult female mealybugs were exposed at 36 °C, and the lowest value was observed when 3rd instar nymphs were exposed at 21 °C (Fig. 1).

### Offspring sex ratio

**Figure 2:**
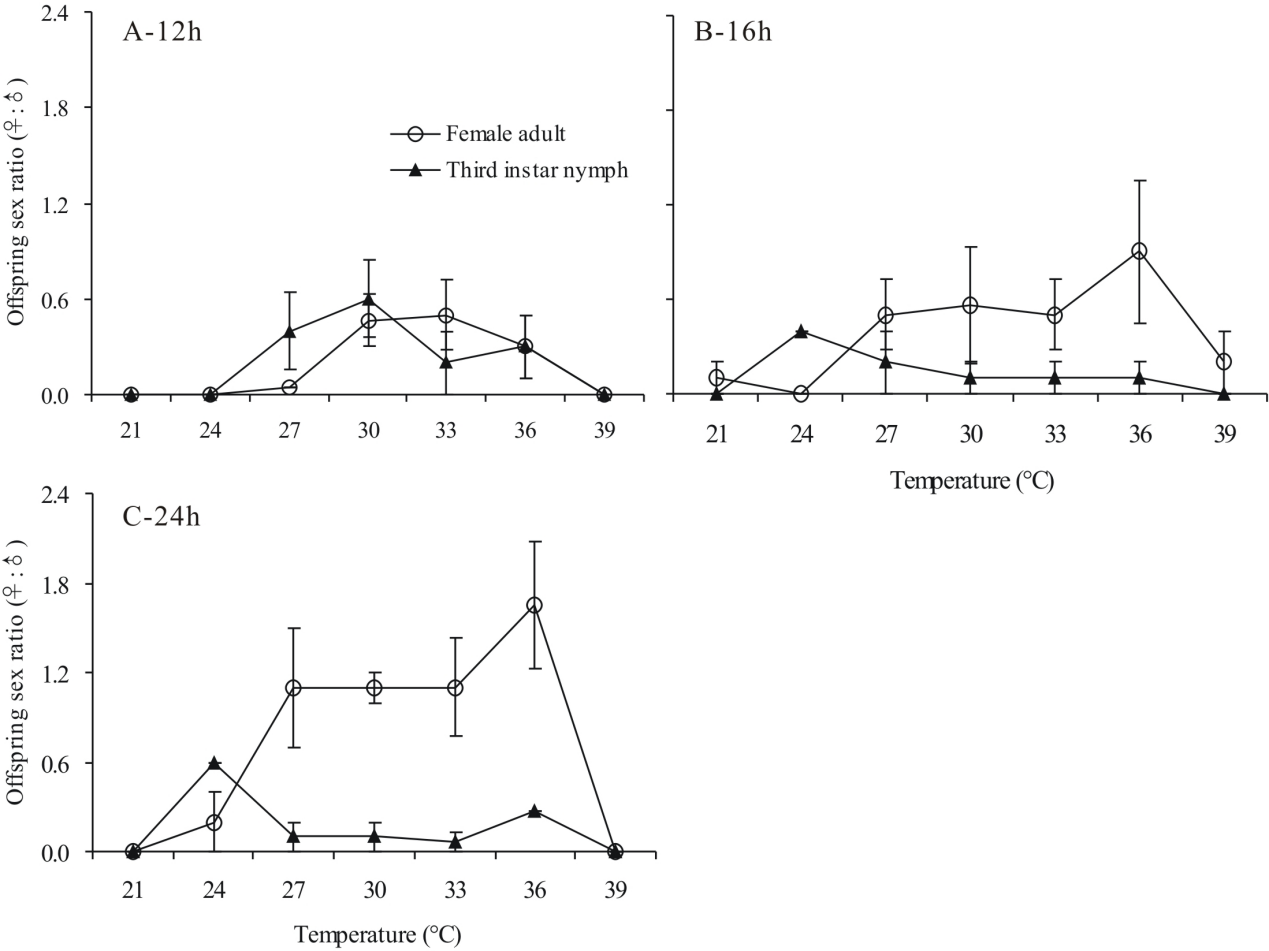
Effects of temperature and host stage on parasitisation and offspring sex ratio of *Aenasius bambawalei* Hayat in *Phenacoccus solenopsis* Tinsley Offspring sex ratio of *Aenasius bambawalei* exposed to temperatures of 21–39 °C for 12 h (A), 16 h (B), and 24 h (C).

In addition to the parasitization rate, the offspring sex ratio is another critical index of parasitic fitness. Therefore, we evaluated the effect of temperature on the offspring sex ratio of *A. bambawalei*. No obvious pattern was observed when wasps were heat-treated for neither 4 h or 8 h; therefore, we did not illustrate these cases. In the case of 12 h and 16 h of exposure, the effect of temperature and host stage was not obvious. However, in the case of 24 h of exposure, both temperature and host stage had significant effects on the offspring sex ratio of *A.bambawalei* (Fig. 2 and Table 2). Further pairwise comparisons indicated that the offspring sex ratio at 30, 33, and 36 °C were significantly higher, and all peaked at 24 h of duration (Fig. 2). *A. bambawalei* exposed to 36 °C for 24 h, showed an offspring sex ratio that dramatically increased by 81.78% compared with those exposed to 12 h. In addition, the sex ratio increased by 45.34% compared with those exposed to 16 h. The offspring sex ratio was clearly higher when the host stage was an adult female mealybug (*t* = 3.862, *P* = 0.018). The value obtained when exposed to 36 °C for 24 h was 1.65 compared with a value of nearly 0 when 3rd instar nymphs were the host. Nevertheless, these results suggest that the data obtained at 27 °C were relatively lower than those obtained by Fand, Gautam & Suroshe (2011) and Abdin et al. (2012b).

A significant interaction (*P* < 0.05) between temperature and host stage was also found (Table 2). The highest and the lowest offspring sex ratio were detected when an adult female was exposed to 36 °C, and a 3rd instar nymph was exposed to 21 °C, respectively (Fig. 2).

**Table 2:** Two-way analysis of variance testing the effect of temperature and host stage on the offspring sex ratio of *Aenasius bambawalei*.

Duration	Source	d*f* (numerator, denominator)	Mean squares	*F*	*P*
12 h	T	6, 69	0.4393	3.891	<0.05
	H	1, 69	0.0194	0.172	0.6797
	T × H	6, 69	0.1083	0.96	0.4609
16 h	T	6, 69	0.3115	1.502	0.1944
	H	1, 69	0.9921	4.783	<0.05
	T × H	6, 69	0.3671	1.77	0.1221
24 h	T	6, 69	0.9831	4.693	<0.01
	H	1, 69	6.6651	31.817	<0.01
	T × H	6, 69	1.3645	6.514	<0.01

**Notes.**

T Temperature H Host stage T × H Temperature × Host stage

## Discussion

Natural enemies are exposed to environmental differences, such as the surrounding temperature, which may affect their biological traits (Fand & Suroshe, 2015). However, most previous experimental studies about the parasitization rate and offspring sex ratio of *A. bambawalei* were conducted under constant temperature (Fand, Gautam & Suroshe, 2011; Abdin et al., 2013). In this study, we tested the effects of different temperatures on the parasitization rate and offspring sex ratio of *A. bambawalei* under laboratory conditions. We found that the parasitization rate increased with higher temperatures and peaked at 30–36 °C after 24 h of exposure. This finding confirmed that environment changes affected the parasitic fitness (Kedar, Saini & Pala, 2012; Nagrare et al., 2011). The host stage is another important factor that affects the fitness of parasitoids’ progeny (Hågvar & Hofsvang, 1991). The parasitization rate of an adult female exposed to high temperatures during 24 h was significantly higher than that of 3rd instar nymphs. A similar finding was also reported by Abdin et al. (2012b), which suggest that larger hosts might result in superior parasitoid fitness (Charnov et al., 1981; Liu, 1985; Harvey, Harvey & Thompson, 1994). However, it was observed that the parasitization rate of *A. bambawalei* in adult mealybugs was lower than that in third instar nymphs (Fand, Gautam & Suroshe, 2011; Abdin et al., 2013) because their defensive behaviors resulted in an increased handling time (Bertschy et al., 2000). In our study, an interaction between temperature and host stage was found, and this interaction possibly was caused by the parasitization rate change. Moreover, this interaction effect only appeared over longer durations (12 h, 16 h, and 24 h), and no difference was detected when the exposure time was shorter (4 h and 8 h). Our results indicate that *A. bambawalei* was adaptable to high temperatures. Likewise, our results are consistent with previous studies on *Oomyzus sokilowskii* (Kurdjumov: Hymenoptera: Eulophidae), which is a gregarious larval-pupal parasitoid of *Plutella xylostella* (Lepidoptera: Plutellidae) (Zhang et al., 2012). A temperature rise led to higher metabolic rates and higher foraging rate of most insects (Wu et al., 2011). Moreover, certain female parasitic wasps can mate immediately after emergence, while males take several hours to become sexually mature (Roux et al., 2010). In addition, the ambient temperature in the earliest ontogenesis could irreversibly determine the sex ratio (Valenzuela, Adams & Janzen, 2003). Hence, the temperature might be related to the sexual maturity of male *A. bambawalei*. To our knowledge, this paper is the first one to investigate the effects of temperature on the parasitization rate and offspring sex ratio of this wasp. However, there are still certain limitations in this study. Although our conclusion has been supported by strong experimental results, this short-term experiment could not fully reveal the effect of temperature. Therefore, we will consider a longer exposure time and temperature-variant environments in future experiments.

Similar to the effect of temperature and host stage on the parasitization rate, the offspring sex ratio was higher when the adult female mealybug was hosted at 30, 33, and 36 °C exposure. This result was consistent with the findings of Abdin et al. (2012b),but it contradicts those of Fand, Gautam & Suroshe (2011). The difference in the ratio of parasitic wasps to mealybugs, and the parasitization time may have led to the differences with previous studies. When our experiment tested 24 h of exposure as reported in previous studies, the offspring sex ratio was stable during this priod. It remains unclear how a longer exposure time would affect the offspring sex ratio. This hypothesis will be tested in the future. Interestingly, the ratio of the offspring female ratio from parasitized adult or nymph was relatively lower than in previous data. Considering that the parasitic fitness of *A. bambawalei* depends on the host on which the mealybug is fed (Nagrare et al., 2011), we will use potato instead of cotton in future experiments. Moreover, in our study, we introduced two pairs of mated *A. bambawalei*, and interference between these two pairs of wasps might also occur (Mohamad, Monge & Goubault, 2013). The results of this study could be applied for the manually massive rearing and release of *A. bambawalei*. In the future, further experiments will be conducted to fully assess the roles of temperature and host stages in suppressing the mealybug population.

## Conclusion

In conclusion, the host stages affected the parasitization rate and offspring sex ratio of *A. bambawalei* under heat stress, which can, in turn, affect the host stage preference of *A. bambawalei*. Therefore, heat stress might ultimately affect the reproductive capacity of *A. bambawalei*. In the future, higher temperatures (higher than 27 °C) will be adopted for the manual massive rearing and releases of this wasp.

## Supplemental Information

10.7717/peerj.1586/supp-1 Supplemental Information 1 Effect of temperature and host stage on parasitization rate

10.7717/peerj.1586/supp-2 Supplemental Information 2 Effect of temperature and host stage on offspring sex ratio

## Additional Information and Declarations

### Data Availability

The following information was supplied regarding data availability: The raw data has been supplied as Supplemental Dataset files.
